# Knowledge, attitudes, and practices towards COVID-19 among Venezuelans during the 2020 epidemic: An online cross-sectional survey

**DOI:** 10.1371/journal.pone.0249022

**Published:** 2021-04-15

**Authors:** Benjamin R. Bates, Adriana Tami, Ana Carvajal, Mario J. Grijalva

**Affiliations:** 1 School of Communication Studies, Ohio University, Athens, OH, United States of America; 2 Department of Biomedical Sciences, Infectious and Tropical Disease Institute, Heritage College of Osteopathic Medicine, Ohio University, Athens, OH, United States of America; 3 Department of Medical Microbiology and Infection Prevention, University Medical Center Groningen, University of Groningen, Groningen, The Netherlands; 4 Facultad de Ciencias de la Salud, Universidad de Carabobo, Valencia, Venezuela; 5 Postgrado de Infectología, Hospital Universitario de Caracas, Caracas, Venezuela; 6 Facultad de Medicina, Universidad Central de Venezuela, Caracas, Venezuela; 7 Centro de Investigación Para la Salud en América Latina, Facultad de Ciencias Exactas y Naturales, Pontificia Universidad Católica del Ecuador, Quito, Ecuador; Iwate Medical University, JAPAN

## Abstract

**Background:**

COVID-19 threatens health systems worldwide, but Venezuela’s system is particularly vulnerable. To prevent the spread of COVID-19, individuals must adopt preventive behaviors. However, to encourage behavior change, we must first understand current knowledge, attitudes, and practices (KAPs) that inform response to this health threat.

**Methods:**

We explored KAPs among Venezuelans using a cross-sectional, internet-based questionnaire. The questionnaire explored individuals’ knowledge about COVID-19; their attitudes toward the world’s and the Venezuelan authorities’ abilities to control it; and their self-reported practices. We also collected demographic data. Binomial logistic regression analyses were used to predict the adoption of preventive behaviors based on demographic variables, individual knowledge level, and individual attitudes.

**Results:**

3122 individuals completed the questionnaire. Participants had a high level of knowledge about COVID-19. They expressed high levels of optimism that the world would eventually control COVID-19, but they were very pessimistic about the public authorities in Venezuela. Most participants adopted preventive practices. Binomial regression suggests younger people, less educated people, and manual laborers hold lower levels of knowledge, and these groups, as well as men, were less likely to adopt preventive practices. Knowledge, by itself, had no association with optimism and little association with self-reported practices.

**Conclusions:**

As other KAP studies in Latin America found, knowledge is not sufficient to prompt behavior change. Venezuelans’ pessimism about their own country’s ability should be explored in greater depth. Health promotion in Venezuela may wish to target the most at risk groups: men, younger people, less educated people, and manual laborers.

## Introduction

Infections with the SARS-CoV-2 virus, and the Coronavirus disease of 2019 (COVID-19) it causes, have spread around the world. COVID-19, given its transmissibility and potential to progress to severe respiratory failure, has the potential to overwhelm healthcare resources [1–4]. Although health systems worldwide are at risk of becoming overwhelmed, because of chronic burden and insufficient funding, healthcare systems in Latin America are particularly vulnerable to collapse [5, 6].

Of all health systems in Latin America, Venezuela’s health care system may be most vulnerable. As reported in *The Lancet* [7], “The healthcare system has all but collapsed,” owing to ongoing challenges of a food crisis, an economy in disarray, a lack of epidemiological data, and the destruction of laboratories across the country. Hospitals face shortages of basic medical supplies, disruptive water and power outages, and flight of healthcare and public health personnel [8–10]. In the five years before the pandemic, nearly half of Venezuela’s health care workers left the country [11]; those who remain are severely underpaid, overworked, and largely unprotected from COVID-19 [12]. Although these challenges pre-date COVID-19 in Venezuela, the pandemic has exacerbated them all: food insecurity has grown more severe as food production and trade have been depressed by the pandemic and as curfews have prevented going out for purchasing food [13]; the economy has shrunk by at least one-quarter domestically and international remittances from Venezuelans living abroad has declined by half [14, 15]; and, domestic civil society organizing and international aid have become even more restricted [16]. These challenges, on their own and as social determinants of health, heighten the threat of COVID-19 to Venezuela.

Within this environment of scarcity, Venezuela has been challenged by a resurgence of infectious diseases like measles, malaria, and diphtheria [17–19]. This resurgence [20] and the potential for these diseases to become syndemic with COVID-19 [21], threatens also the health of people throughout the region given the spillover of diseases driven by mass migration out of Venezuela. Health systems of neighboring countries especially, have been burdened by the number of Venezuelan migrants associated with the spill-over of other infectious diseases including malaria, measles, and Zika [22, 23]. COVID-19 could follow a similar pattern [21].

Venezuela is unlikely to have the resources to effectively respond to COVID-19. Moreover, there is no currently available vaccine or standardized treatment regimen [24]. Although Venezuela claims to have among the lowest incidence of COVID-19 in Latin America, with official reports of a total 117.000 cases and 1.100 deaths as of January 10, 2021 [25], these numbers are likely to be significantly deflated [26]. Journalists [27, 28], scientists [29, 30], and opposition politicians [31, 32] report higher numbers, even in the face of arrest and punishment. Moreover, the Venezuelan government has chosen to use tests with higher rates of false negatives than recommended, and even these tests are in short supply [33, 34]. As reported by Human Rights Watch, “The real number is almost certainly much higher, given the limited availability of reliable testing, limited transparency, and the persecution of medical professionals and journalists who report on this issue” in Venezuela [35].

Given an extremely unstable health care system, limited resources, and a problem of unknown but large magnitude, primary prevention of exposure to SARS-CoV-2 is essential [36–38]. These strategies include hand hygiene, mask-wearing, and avoidance of public contact [39, 40]. In infectious disease contexts, adoption of preventive strategies depends on people’s knowledge, attitudes, and practices (KAPs) related to the health threat [41–45]. To better promoting these primary prevention practices, we explored KAPs related to COVID-19 among the Venezuelan public.

## Materials and methods

### Participants

The data for this study were collected online from July 1 to July 14, 2020 using the Qualtrics platform. Participants were recruited through the authors’ personal networks inviting persons in Venezuela to forward an e-mail recruitment message and WhatsApp messages were sent to the authors’ personal networks with a request to share. Approximately fifty seed messages were sent out by email and WhatsApp. In addition, participants were recruited through one paid social media post on each of Facebook and Twitter with a link to the survey. Social media posts were only shown to individuals who had set their residence as in Venezuela. Paid social media posts were set to run until 10.000 clicks were attained or one week had expired. People who identified as Venezuelans and who were aged 18 or greater were asked to click a link that brought them to the first study page.

Participants were informed about the purpose of the study, on the time and risk the questionnaire involved, and assured of anonymity. Participants were asked to confirm their voluntary willingness to participate and that they were at least 18 years old. This study protocol was approved by the Institutional Review Board at Ohio University (20-E-156) as an exempt study with a waiver of signed informed consent. As of 2020 Venezuela research review regulations only apply to experimental biological or medical research; social/behavioral research did not need ethics review.

### Measures

The KAP questionnaire was based on Zhong and colleagues’ instrument designed for the People’s Republic of China [46] as it was adapted by Bates et al [47] for Latin American contexts. We adapted the questionnaire to the Venezuelan context, such as a changing the options for civil status, accounting for documented and undocumented Venezuelans, and altering the list of cities of residence. The knowledge index, as well as the attitude and practice items, was validated by medical and public health experts in the original Zhong study and, for the current study specifically, by medical and public health experts residing in Venezuela.

The questionnaire began with demographic variables. These were age, gender, marital status, education level, occupation, and country of current residence. If a participant indicated residence in Venezuela, they were provided a list of the five largest cities in Venezuela, an open-ended option to name another large city, or an option to choose living outside of a city. If they indicated residence outside of Venezuela, they were asked to name where they resided (a list of other countries in Latin America, the USA, the Caribbean, Europe, Africa, or other).

Following the demographic questions, participants were presented with KAP items.

Knowledge of COVID-19 was assessed with 12 questions. Four questions addressed knowledge of the clinical presentation of COVID-19, three addressed transmission pathways, and five addressed efficacy of prevention and control measures. Participants could answer “true”, “false”, or “don’t know”. Correct answers were assigned 1 point; other answers were assigned 0 points. Points were summed for a total knowledge score of 0–12, with higher scores indicating better knowledge of COVID-19.

Two questions assessed attitudes. The first question asked whether the participants agreed that COVID-19 will ultimately be controlled globally, disagreed, or didn’t know if they agreed or disagreed. The second asked whether the participants had confidence or not that Venezuela’s government would win the battle against COVID-19.

Three items assessed practices associated with preventive strategies for the spread of COVID-19. We asked participants to self-report whether they avoided crowded places, wore masks when they left the home, and washed their hands when they returned home or had been in contact with other people. If a person stated they had left their home in the past week, we also asked them the number of times they left home, why they had left, and whether they maintained a distance of at least 2 meters from other people.

### Statistical procedures

All data analyses were conducted with SPSS version 26.0. First, frequencies of correct knowledge answers and various attitudes and practices were described (see Table 1). Then, to compare members of different demographic groupings’ knowledge scores, attitudes, and practices, independent-samples t-tests, one-way analyses of variance (ANOVA), and Chi-Square tests, as appropriate, were used as initial tests. Binary logistic regression analyses were then used to identify demographic factors associated with each attitude and each practice. Odds ratios (ORs), and their 95% confidence intervals (CIs), were used to assess the associations between demographic variables and KAP. Following best sample size practices for binomial logistic regression, because the subset of the data from persons who had left the home in the last week did not meet sample size requirements, we reported frequencies only for this subset and not differences within that subset. The statistical significance level was set at p < .05.

**Table 1:** Knowledge, attitudes, and practice towards COVID-19.

Questions	Correct rate, % of total sample endorsing	Options
K1.The main clinical symptoms of COVID-19 are fever, fatigue, dry cough, and muscle pain.	90.2%	True, False, Don’t Know
K2. Unlike the common cold, stuffy nose, runny nose, and sneezing are less common in persons infected with COVID-19.	44.2%	True, False, Don’t Know
K3. There currently is no effective cure for COVID-19, but early symptomatic and supportive treatment can help most patients recover from the infection.	91.4%	True, False, Don’t Know
K4. Not all persons with COVID-2019 will develop to severe cases. Those who are elderly, have chronic illnesses, and are obese are more likely to be severe cases.	92.7%	True, False, Don’t Know
K5. Taking antibiotic medications helps to prevent and treat COVID-19 (reverse coded)	66.8%	True, False, Don’t Know
K6. Persons with COVID-19 can only transmit the virus to other people when they have a fever (reverse coded)	86.1%	True, False, Don’t Know
K7. COVID-19 spreads via respiratory droplets of infected individuals.	90.9%	True, False, Don’t Know
K8. Ordinary citizens can wear general medical masks to prevent infection by the COVID-19 virus.	83.2%	True, False, Don’t Know
K9. It is not necessary for children and young adults to take measures to prevent the infection by the COVID-19 virus (reverse coded).	93.4%	True, False, Don’t Know
K10. To prevent COVID-19, individuals should avoid going to crowded places such as bus stations and avoid taking public transportation.	97.2%	True, False, Don’t Know
K11. Isolation and treatment of people who have COVID-19 are effective ways to reduce the spread of the virus.	96.9%	True, False, Don’t Know
K12. People who have contact with someone infected with the COVID-19 virus should be immediately isolated in a proper place. In general, the observation period is 14 days	96.3%	True, False, Don’t Know
A1. Do you agree that COVID-19 will finally be successfully controlled?	63.9%	Yes, No, Don’t Know
A2. Do you have confidence that the public authorities in Venezuela can win the battle against COVID-19?	37.4%	Yes, No
P1. In the past week, have you gone to any crowded place?	24.5%	Yes, No
P2. In the past week, have you worn a mask when leaving home?	92.8%	Yes, No
P3. In the past week, have you washed your hands for at least 20 seconds each time you have returned home or touched another person?	90.1%	Yes, No
P4. Since the beginning of the COVID-19 crisis have you taken an antibiotic medication to treat or prevent COVID-19?	6.7%	Yes, No

## Results

A total of 3870 individuals consented to the survey. Participants who reported either being under 18 years of age (n = 333) or not identifying as being Venezuelan (n = 122) on the screening questions were excluded, and no further data were recorded for them. Participants who reported living outside of Venezuela and/or who did not report an answer on all substantive questions were excluded (n = 293); 3122 individuals were retained. This final sample was majority female (2215, 70.9%). A majority reported being over 50 years of age (1814, 58.1%). About one-third of the participants were from Caracas, the national capital (809, 28.1%), one-fifth reported living outside of large cities (114, 21.6%), and the remainder were from various large cities across Venezuela. About 5% of the sample chose to not answer one or more demographic questions. Full demographic characteristics are shown in Table 2.

**Table 2:** Demographic characteristics and COVID-19 knowledge score differences.

Characteristics		Number of Participants (%)	Knowledge Score (mean ± standard deviation)	t/F	p
Gender	Male	767 (24.6)	10.32 (1.45)		
	Female	2215 (70.9)	10.33 (1.45)	-0.15	0.90
	Other†	18 (0.6)	--		
	No report†	122 (3.9)			
Age-Grouping	18–29	231 (7.4)	10.09 _a_ (1.63)		
	30–49	911 (29.2)	10.27 _ab_ (1.56)		
	50+	1814 (58.1)	10.38 _b_ (1.38)	5.12	<0.01
	No report†	266 (5.3)	--		
Marital Status	Single-Never Married	620 (19.9)	10.37_abd_ (1.44)		
	Married	1212 (38.8)	10.43_abd_ (1.37)		
	Separated	183 (5.9)	10.14_acd_ (1.44)		
	Divorced	368 (11.8)	10.23_abd_ (1.47)		
	Widowed	203 (6.5)	10.23_abd_ (1.57)		
	Cohabitating	397 (12.7)	10.09_cd_ (1.62)	4.51	<0.01
	No report†	139 (4.5)	--		
Education	Elementary	52 (1.7)	9.00_a_ (2.45)		
	Secondary	695 (22.3)	9.94_b_ (1.74)		
	Bachelor’s Degree	1265 (40.5)	10.34_c_ (1.35)		
	Master’s Degree or Higher	967 (31.0)	10.67_d_ (1.14)	50.65	< .01
	No report†	143 (4.6)	--		
Occupation	Manual Labor	183 (5.9)	9.97_a_ (1.84)		
	Office Work	487 (15.6)	10.41_b_ (1.36)		
	Sales or Service	353 (11.3)	10.27_bc_ (1.52)		
	Education Sector	439 (14.1)	10.23_abc_ (1.31)		
	Health Sector	526 (16.8)	10.86d (1.10)		
	Student	60 (1.9)	10.33_abc_ (1.28)		
	Housewife/Househusband	577 (18.5)	10.11_ac_ (1.55)		
	Unemployed	342 (11.0)	10.12_ac_ (1.62)	15.33	< .01
	No report†	155 (5.0)	--		
Residence‡	Caracas	813 (26.0)	10.38_ace_ (1.47)		
	Maracaibo	175 (5.6)	10.03_be_ (1.60)		
	Valencia	293 (9.4)	10.42_acde_ (1.27)		
	Barquisimeto	153 (4.9)	10.27_acde_ (1.66)		
	Maracay	178 (5.7)	10.57_acde_ (1.15)		
	Other Large City	655 (21.0)	10.34_acde_ (1.44)		
	Not in a Large City	625 (20.0)	10.26_bcde_ (1.45)	2.67	< .02
	No report†	230 (7.4)	--		

Notes: Participants who did not report excluded, totals may not add to 3122 within demographic groupings; t/F indicates t values between two groups in independent sample T-tests or F values for ANOVA tests; p indicates level of statistical significance; means with different subscripts differ at p < .05 level;

† excluded from further analysis because test assumptions violated;

‡ participants living outside of Venezuela excluded

The correct answer rates of the 12 questions on the COVID-19 knowledge questionnaire ranged from poor (with only 44.2% answering correctly that symptoms of the common cold may differ from COVID-19) to near universal knowledge (with 97% answering correctly that avoiding crowded places and that isolation and treatment are important to reducing transmission of the virus). Table 1 reports correct answer rates for each item. The mean COVID-19 knowledge score was 10.29 (SD: 1.50, range: 0–12), suggesting a relatively high rate of knowledge. Knowledge scores significantly differed by age group, with older individuals having more COVID-19 related knowledge, and by educational status, with an apparently linear relationship (see Table 2). Knowledge scores differed by marital status, with cohabitating persons scoring lowest and single and non-divorced ever-married persons scoring highest. Knowledge scores also differed by employment status, with manual laborers scoring lower, and individuals who work in health and medicine scoring highest. Persons living in Maracaibo scored significantly lower than individuals who lived in Venezuela’s other large cities or in non-urban areas. Gender was not associated with differences in levels of knowledge.

Respondents were generally optimistic about the eventual successful control of COVID-19. About two-thirds of the sample (n = 1995; 63.9%) agreed that COVID-19 will eventually be successfully controlled globally, while only one-tenth (n = 317; 10.2%) disagreed. The remainder (n = 803; 25.7%) stated that they did not know. Respondent’s attitudes toward final success differed significantly by gender, age grouping, educational level, occupational status, and place of residence in the initial analyses (see Table 3). Overall, the binary logistic regression model successfully classified 86.3% of cases and explained about 6% of all variance in approaching or avoiding crowded places (Nagelkerke R2 = .06 “Don’t Know” excluded). Binary logistic regression revealed that older participants were more likely to believe successful control will eventually be attained (middle group as compared to youngest group, OR 1.60 (0.92, 2.79), p < .01; oldest group as compared to younger groups, OR 1.61 (1.20, 2.164), p < .01). Persons who worked in the health sector as compared to all other groups were more likely to predict the world’s success, OR 2.38 (1.40, 4.03), p < .01), as were students when compared to all other groups , OR 2.40 (0.98, 5.92), p < .05). Although preliminary Chi-square analyses indicated significant differences by gender and by educational level and preliminary t-tests indicated significant differences in knowledge, they did not emerge as significant predictors in the regression model.

**Table 3:** Attitudes towards COVID-19 by demographic variables.

Characteristics		Attitudes, n (%) or mean (s.d.)				
		A1. Ultimate Success in Controlling			A2. Confidence of Winning in Venezuela	
		Agree	Disagree	Don’t Know	Yes	No
Gender	Male	555 (72.4%)	80 (10.4%)	132 (17.2%)	297 (389.9%)	466 (61.1%)
	Female	1364 (61.7%)	223 (10.1%)	623 (28.2%)**	821 (37.8)	1353 (62.2%)
Age-Grouping	18–29	134 (58.0)	30 (10.0)	67 (29.0)	78 (34.2)	150 (65.8)
	30–49	552 (60.8)	114 (12.6)	242 (26.7)	351 (39.2)	545 (60.8)
	50+	1213 (67.0)	157 (8.7)	441 (24.4)**	671 (37.6)	1115 (62.4)
Marital Status	Single-Never Married	372 (60.0)	70 (11.3)	178 (28.7)	238 (39.0)	372 (61.0)
	Married	791 (65.4)	126 (10.4)	293 (24.2)	414 (34.8)	777 (65.2)
	Separated	115 (62.8)	22 (12.0)	46 (25.1)	74 (41.1)	106 (58.9)
	Divorced	249 (68.0)	30 (8.2)	87 (23.8)	126 (34.7)	237 (65.3)
	Widowed	124 (61.7)	20 (10.0)	57 (28.34)	76 (38.2)	123 (61.8)
	Cohabitating	264 (66.5)	37 (9.3)	96 (24.2)	180 (45.7)	214 (54.3)**
Education	Elementary	30 (60.0)	0 (0.0)	20 (40.0)	27 (54.0)	23 (46.0)
	Secondary	473 (68.1)	55 (7.9)	167 (24.0)	323 (47.0)	364 (53.0)
	Bachelor’s Degree	796 (63.1)	131 (10.4)	334 (26.5)	453 (36.5)	788 (63.5)
	Master’s Degree or Higher	613 (63.4)	119 (12.3)	235 (24.3)**	304 (31.8)	651 (68.2)**
Occupation	Manual Labor	123 (67.2)	15 (8.2)	45 (24.6)	77 (42.5)	104 (57.5)
	Office Work	323 (66.5)	46 (15.1)	117 (24.1)	187 (39.0)	293 (61.0)
	Sales or Service	225 (63.7)	39 (11.0)	89 (25.2)	137 (39.1)	213 (60.9)
	Education Sector	300 (68.3)	46 (10.5)	93 (21.2)	182 (41.8)	253 (58.2)
	Health Sector	294 (55.9)	88 (16.7)	144 (27.4)	156 (30.1)	362 (69.9)
	Student	34 (56.7)	11 (18.3)	15 (25.0)	23 (39.0)	36 (61.0)
	Housewife/Househusband	368 (64.1)	33 (5.7)	173 (30.1)	221 (39.5)	338 (60.5)
	Unemployed	235 (69.1)	26 (7.6)	79 (23.2)**	122 (36.0)	217 (64.0)**
Residence	Caracas	486 (59.6)	79 (9.7)	249 (30.7)	282 (35.2)	519 (64.8)
	Maracaibo	117 (66.9)	15 (8.6)	43 (24.6)	68 (39.5)	104 (60.5)
	Valencia	177 (60.4)	37 (12.6)	79 (27.0)	84 (29.2)	204 (70.8)
	Barquisimeto	102 (66.7)	14 (9.2)	37 (24.2)	60 (39.5)	92 (60.5)
	Maracay	116 (65.2)	15 (8.4)	47 (26.4)	71 (40.3)	105 (59.7)
	Other Large City	454 (69.4)	59 (9.0)	141 (21.6)	288 (44.2)	364 (55.8)
	Not in a Large City	413 (66.1)	77 (12.3)	135 (21.6)**	233 (38.1)	379 (61.9)**
Knowledge		10.31 (1.48)	10.16 (1.77)	10.31 (1.43)	10.28 (1.46)	10.32 (1.50)

Notes: Participants who did not report excluded, totals do not add to 2,399 within demographic groupings; † Other Large Cities are all self-reported cities with fewer than 20 respondents naming that city;

* Chi-square values significant at p < .05;

** Chi-square values significant at p < .01; † Don’t Know differs from other groups at p < .01

Unlike their view regarding the global situation, participants reported much more pessimism about whether the public authorities in Venezuela would be successful in controlling COVID-19. In a near reversal of their opinions about the world’s ability to control COVID-19, about two-thirds of participants (n = 1899; 60.8%) did not have confidence that Venezuela’s public authorities would succeed, while the remainder did have confidence in the public authorities (n = 1169; 37.4%). Respondent’s attitudes toward Venezuela’s success differed significantly by civil status, educational level, occupation type, and place of residence (see Table 3). Overall, the binary logistic regression model successfully classified 62.7% of cases and explained about 5% of all variance in attitudes to toward Venezuela’s winning the battle against COVID-19 (Nagelkerke R2 = .51). Binary logistic regression revealed that participants who were middle-aged were more likely to predict Venezuela’s failure to control COVID-19 as compared to all other age groups, OR 1.46 (1.02, 2.08), p < .05. As participants were better educated, they were more likely to predict Venezuela’s inability to control COVID-19 (secondary completers as compared to primary school completers, OR 0.29 [.15, .56], p < .01; bachelor’s degree holders compared to less educated groups, OR 0.48 [.37, .61], p < .01; master’s degree or higher completers as compared to less educated groups, OR .81 [.66, .99], p < .01). Workers in the health sector were more likely to predict failure as compared to all other occupations, OR .98 (.71, 1.35), p < 01. People living in Caracas, OR 1.18 (.94, 1.47), p < .01, or living in Valencia, OR 1.60 (1.64, 2.19), p < .01, were more likely to predict Venezuela losing the battle against COVID-19 as compared to residents of other places.

In the realm of practices, most participants stated that they followed approved practices. Most participants (n = 2354, n = 75.4%) stated that they have not gone to a crowded place in the last week. Respondent’s self-report that they had avoided crowded places differed significantly by gender, age grouping, civil status, and occupation type in the initial comparisons (see Table 4). Overall, the binary logistic regression model successfully classified 74.3% of cases and explained about 6.4% of all variance in approaching or avoiding crowded places (Nagelkerke R2 = .064). Binary logistic regression revealed that men were less likely to have avoided crowded places than women (OR: 0.70 (.57, .86); p < .05). As persons were older, they were more likely to have avoided crowded places (youngest group as compared to middle group, OR .39 (0.27, 0.55), p < .01; younger groups as compared to oldest groups, OR .64 (.53, .79), p < .01). Homemakers were best able to avoid crowded places compared to all other groups, OR 2.21 (1.09, 4.47), p < .05. Persons who were more knowledgeable about COVID-19 were also better able to avoid crowded places, OR 1.07 (1.01, 1.14), p < .05. Although preliminary comparisons analyses indicated significant differences by civil status, civil status did not emerge as a significant predictor in the regression model.

**Table 4:** COVID-19 Control Practices by demographic variables.

Characteristics		Practices, n (%) or mean (s.d.)							
		P1. Going to Crowded Places		P2. Wearing a Mask		P3. Handwashing		P4. Taking Preventive Antibiotics	
		Yes	No	Yes	No	Yes	No	Yes	No
Gender	Male	231 (30.2)	535 (69.8)	724 (98.4)	12 (1.6)	696 (91.1)	98 (8.9)	42 (5.5)	721 (94.5)
	Female	510 (23.0)	1704 (77.0)*	2113 (98.4)	34 (1.6)	2052 (93.1)	53 (6.9)	81 (3.7)	2126 (96.3)*
Age-Grouping	18–29	88 (38.1)	143 (61.9)	218 (97.8)	5 (2.2)	189 (83.3)	38 (16.7)	16 (7.0)	211 (93.0)
	30–49	278 (30.5)	633 (69.5)	887 (98.2)	16 (1.8)	837 (92.2)	71 (7.8)	42 (4.6)	866 (95.4)
	50+	373 (20.6)	1438 (79.4)*	1713 (98.7)	23 (1.3)	1699 (94.1)	2725 (92.7)*	62 (3.54)	1747 (96.6)*
Marital Status	Single-Never Married	192 (31.0)	428 (69.0)	594 (98.5)	9 (1.5)	559 (90.9)	56 (9.1)	38 (6.2)	578 (93.8)
	Married	268 (22.1)	944 (77.9)	1147 (98.5)	18 (1.5)	1142 (94.5)	66 (5.5)	45 (3.7)	1163 (96.3)
	Separated	49 (26.8)	134 (73.2)	170 (96.6)	6 (3.4)	159 (87.4)	12 (12.6)	2 (1.1)	181 (98.9)
	Divorced	90 (24.7)	275 (75.3)	355 (98.9)	4 (1.1)	344 (94.0)	22 (6.0)	16 (4.4)	350 (95.6)
	Widowed	30 (14.8)	173 (85.2)	184 (97.4)	5 (2.6)	189 (93.6)	13 (6.4)	3 (1.5)	200 (98.5)
	Cohabitating	110 (27.7)	287 (72.3)*	384 (99.0)	4 (1.0)	355 (89.9)	40 (10.0)*	18 (4.6)	377 (95.4)*
Education	Elementary	14 (26.9)	38 (73.1)	48 (98.0)	1 (2.2)	46 (90.2)	5 (9.8)	5 (9.6)	47 (90.4)
	Secondary	159 (22.9)	536 (77.1)	652 (98.3)	11 (1.7)	629 (91.4)	59 (8.6)	31 (4.5)	659 (95.5)
	Bachelor’s Degree	335 (26.5)	928 (73.5)	1203 (98.4)	19 (1.6)	1167 (92.5)	95 (7.5)	55 (4.4)	1206 (95.6)
	Master’s Degree or Higher	231 (23.9)	735 (76.1)	929 (98.5)	14 (1.5)	903 (93.8)	60 (6.2)	30 (3.1)	934 (96.9)
Occupation	Manual Labor	50 (27.3)	133 (72.7)	173 (99.4)	1 (0.6)	160 (8739)	22 (12.1)	16 (8.8)	166 (91.2)
	Office Work	140 (28.7)	347 (71.3)	473 (99.0)	5 (1.0)	458 (94.2)	28 (5.8)	16 (3.3)	471 (96.7)
	Sales or Service	112 (31.7)	241 (68.3)	337 (97.7)	8 (2.3)	321 (90.9)	32 (9.1)	26 (7.4)	326 (92.6)
	Education Sector	85 (19.4)	353 (80.6)	414 (98.3)	7 (1.7)	407 (92.9)	31 (7.1)	16 (3.7)	421 (96.3)
	Health Sector	157 (29.8)	369 (70.2)	510 (99.2)	4 (0.8)	500 (95.6)	23 (4.4)	12 (2.3)	512 (97.7)
	Student	14 (23.3)	46 (76.7)	54 (94.7)	3 (5.3)	49 (84.5)	9 (15.5)	4 (6.9)	54 (93.1)
	Housewife/ Househusband	98 (17.0)	478 (83.0)	541 (98.2)	10 (1.8)	529 (92.5)	43 (7.5)	16 (2.8)	558 (97.2)
	Unemployed	81 (23.8)	260 (76.2)*	316 (97.5)	8 (2.5)	308 (90.6)	32 (9.4)*	16 (4.7)	325 (95.3)*
Residence	Caracas	202 (24.9)	610 (75.1)	776 (98.9)	9 (1.1)	768 (95.0)	40 (5.0)	30 (3.74)	779 (96.3)
	Maracaibo	43 (24.6)	132 (75.4)	163 (98.8)	2 (1.2)	160 (92.5)	13 (7.5)	22 (12.6)	152 (87.4)
	Valencia	74 (25.3)	219 (74.7)	280 (99.3)	2 (0.7)	275 (93.9)	18 (6.1)	6 (2.0)	287 (98.0)
	Barquisimeto	34 (22.2)	119 (77.8)	143 (97.9)	3 (2.1)	137 (90.1)	15 (9.9)	4 (2.6)	148 (97.4)
	Maracay	35 (19.7)	143 (80.3)	170 (97.1)	5 (2.9)	164 (92.1)	14 (7.9)	5 (2.8)	173 (97.2)
	Other Large City	185 (28.3)	469 (71.7)	624 (98.0)	13 (2.0)	602 (92.3)	50 (7.7)	21 (3.2)	630 (96.8)
	Not in a Large City	154 (24.6)	471 (75.4)	591 (98.2)	11 (1.8)	556 (89.4)	66 (10.6)*	29 (4.7)	594 (95.3)*
Knowledge		10.21 (1.61)	10.32 (1.46)	10.34 (1.45)	9.89 (2.15)**	10.35 (1.45)	9.91 (1.66)†	9.80 (1.63)	10.34 (1.46)†

Notes: Participants who did not report excluded, totals do not add to 2,399 within demographic groupings;

† Other Large Cities are all self-reported cities with fewer than 20 respondents naming that city;

* Chi-square values significant at p < .05;

** t values indicate significant difference at p < .01; † t values indicate significant difference at p < .05

Even more encouraging, most participants (n = 2897; 92.8%) reported that they had worn a mask when they had left the home in the last week. Preliminary tests indicated that respondent’s self-report that they had worn a mask whenever they leave the home did not differ significantly by any demographic variable but did by knowledge of COVID-19 (see Table 4). Overall, the binary logistic regression model successfully classified 98.4% of cases and explained about 6% of all variance in mask wearing (Nagelkerke R2 = .062). Binary logistic regression revealed that students were much less likely to wear a mask as compared to all other groups, OR: 0.24 (.06, .98), p < .05). This result should be read with caution, however, as there were fewer cases of mask failure (3) than predictor variables in the regression model Although preliminary t-test analyses indicated significant differences by knowledge of COVID-19, knowledge did not emerge as a significant predictor in the regression model.

An overwhelming majority of participants (n = 2812; 90.1%) stated that they had washed their hands for at least 20 seconds every time after returning home or touching another person. Respondent’s self-report of handwashing practices differed significantly by all demographic variables except gender and educational attainment and differed by knowledge, in the initial comparisons (see Table 4). Overall, the binary logistic regression model successfully classified 92.5% of cases and explained about 7% of all variance in handwashing (Nagelkerke R2 = .073). Binary logistic regression revealed that women were more likely than men to engage in proper handwashing, OR 1.54 (1.10, 2.16), p < .05. As persons were older, they were more likely to properly wash their hands (middle group as compared to youngest group, OR 2.88 (1.71, 4.87), p < .01; oldest group as compared to younger groups, OR 1.33 (.94, 1.87), p < .01). Persons who reported their civil status as separated were least likely to correctly wash their hands as compared to all other civil status, OR .61 (.39, .96), p < .05. Of all occupational groups, students were least likely to wash their hands properly, OR .50 (.27, .92), p < .05) as compared to all other occupations, and persons living in Maracaibo, as compared to all other places of residence, were less likely to correctly wash their hands, OR .47 (.23, .95), p < .05. Finally, persons who held lower levels of knowledge were less likely to correctly wash their hands, OR .87 (.80, .95), p < .01.

Most participants (n = 2913; 93.3%) stated that they had not taken any antibiotic medications to treat or prevent COVID-19. Respondent’s self-report of antibiotics practices differed by all demographic variables except educational attainment and by knowledge in the initial comparisons (see Table 4). Overall, the binary logistic regression model successfully classified 95.9% of cases and explained about 9% of all variance in taking antibiotics (Nagelkerke R2 = .094). Binary logistic regression revealed that persons who had completed only primary school were least likely to take antibiotics compared to all other groups, OR .32 (.10, .98), p < .05). Manual laborers, compared to all other groups, were most likely to take antibiotics for prevention of treatment of COVID-19, OR 1.02 (.47, 2.22), p < .05, while office workers, compared to all other groups, were least likely, OR .51 (.25, 1.04), p < .05). Finally, persons with lower levels of knowledge about COVID-19 were more likely to take antibiotics than were more knowledgeable people, OR 1.16 (1.03, 1.30), p < .05. Although gender, age, and civil status were significant predictors in the initial analyses, they did not emerge as predictors in the regression model.

Most participants stated that they had not left home in the past week (n = 2377; 76.1%). Among those (n = 745, 23.9%) who had left the home in the last week, 205 (27.5%) had left the home once, 307 (41.2%) had left home two or three times, and 233 people (31.3% of those who had left home, 7.5% of the total sample) had left home four or more times. Most people who had left the home reported maintaining a distance of at least two meters from other people (n = 474; 62.9%). When those who had left the home were asked why, they reported going to essential work (n = 300; 40.3%), buying food for self, family, or pets (n = 589; 79.1%), buying medicine (n = 205; 27.5%), or going to the doctor or veterinarian (n = 83; 11.1%). None of the people who had left home reported a non-essential reason for doing so.

## Discussion

This study was conducted during the global COVID-19 pandemic and during a period of rapid rise in positive cases in Venezuela [48, 49]. Although Venezuela is at high risk of health system collapse in the face of COVID-19, both independent scientific [50] and journalistic reporting [51] have been limited by the government. This study of KAP regarding COVID-19 in Venezuela may provide guidance for the Ministry of Popular Power for Health in Venezuela, as well intergovernmental organizations and non-profit and private sector actors, seeking to promote prevention strategies for this disease.

Because COVID-19 is a regional threat in Latin America, identifying similarities and differences between KAPs among different national populations may allow Ministries of Health to learn best practices from other nations or identify unique intervention points for their people. In this instance, similarities and differences in KAPs in Venezuela and in two other Latin American states–Colombia and Ecuador–can be identified because our methodology and sample are similar to KAP investigations previously conducted in Colombia and in Ecuador [47, 52]. Not only are the current study’s demographic characteristics similarly distributed in terms of age, gender, socioeconomic status, and urban/rural residency, but eleven of twelve knowledge items, both attitudinal items, and three of the four practice items are identical to Bates et al.’s studies. These allow direct comparison to be drawn among national populations.

In comparison to Bates et al.’s studies, people in Venezuela are somewhat more knowledgeable about COVID-19. The Venezuelans in this sample answered 85.8% of questions correctly, compared to 76.8% of Colombians and 82.3% of Ecuadorians. Like these other samples in Ecuador and Colombia, younger people, less educated people, and manual laborers hold lower levels of knowledge. Specific areas where knowledge could be improved in Venezuela are the differences in symptoms between the common cold and COVID-19, as well as the inutility of antibiotics in preventing COVID-19 and the utility of wearing a mask. Significantly, where Bates et al.’s [47, 52] previous studies found few associations between knowledge and preventive practices, our data reveals that Venezuelans who are more knowledgeable of COVID-19 are more likely to wash their hands correctly, more likely to wear masks when in public, and less likely to improperly take antibiotics. Because these practices correspond fairly well to knowledge gaps among Venezuelans, they present clear and direct targets for educational messaging, and this kind of scientifically accurate messaging, if properly disseminated, could have a substantial impact on improving healthy practices [53–55].

In addition to promoting knowledge of a disease to aid in supporting preventive practices, it is common for public health advocates to try to create positive attitudes about overcoming health challenges to promote action [56, 57]. In our data, Venezuelans were substantially more likely to believe that the world, as a whole, would overcome COVID-19 than they were to believe the public authorities in Venezuela would be successful. This is likely due to their own experience within the country’s deep economic crises, faltering health system and political turmoil [7–10]. To better enable support of public and private efforts to control COVID-19, organizations may wish to focus on actions that would improve the outlook of persons who have high opinions of the world’s abilities but lower opinions of Venezuela’s abilities to overcome COVID-19. These actions, if undertaken by the government or private sector actors, should be practical, visible, widespread and accompanied by messaging that should include persons of middle age, persons with more education, and, significantly, persons who work in the health sector of the economy, as well as residents of Caracas and Valencia. Without significant improvement of the current conditions, changing the attitudes of persons who have a low estimation of the public health authorities in Venezuela’s ability to overcome COVID-19 may be extremely difficult.

Although the majority of Venezuelans self-report following recommended practices, there is still room for improvement. In particular, nearly one-fourth of Venezuelans report having gone to crowded places in the last week, a number higher than self-reported in both Colombia and in Ecuador [47, 52]. The groups that appear to be less likely to follow approved practices are men, younger people, students, and manual laborers. These groups may suggest particular targets for disease prevention campaigns that emphasize proper preventive practices. Young people, a group that often includes students, and particularly young men, are more likely to express feeling of invincibility to COVID-19 [58], making it essential that public health advice be crafted effectively for them. The other group, manual laborers, may have a reduced ability to adopt preventive practices because of the nature of their work; manual labor cannot be performed remotely and is often done in concert with other manual laborers (making it difficult to avoid crowds) and is strenuous (making mask wearing difficult). To assist manual laborers in adopting proper preventive practices for COVID-19, it may be necessary to focus on improving their work conditions overall rather than focusing on health messaging.

Finally, these messaging strategies must account for the unique communication challenges in Venezuela. The groups most in need of persuasion, particularly individuals of lower socioeconomic status have greater difficulties in accessing mobile phones and the internet [59]; yet, during this pandemic, more information in Venezuela has been shared through social media and on the internet compared to national radio and television broadcast media [60]. This lack of access is worrisome given that Venezuela generally has slow and unstable telecommunications infrastructure [61] and what telecommunications structures that exist in Venezuela are at risk of collapse [60]. Although it is relatively easy to recommend greater transparency and accessibility to epidemiological data is an emergency in complex health situations, as well as to recommend publicizing actions that allow addressing the current health situation [62], it is vital to also assess and address the feasibility of these recommendations given. To fully allow messaging in places like Venezuela, the message must be well designed and must account for the availability and stability of technological and information tools.

### Limitations

This study is the only, to our knowledge, study to examine knowledge, attitudes and practices related to COVID-19 among the general public in Venezuela. Given the restricted research and reporting environment within Venezuela, and given the precarity of Venezuela’s health system, it is essential that we use this information as a starting point for improving communication and intervention campaigns. Nonetheless, there are some limitations to this study. One limitation is that there may be a social desirability bias in the data, in that participants may overreport compliance with recommended preventive behaviors. If the data are biased by socially desirable responding, this would be a systemic bias would be systemic and the binary logistic regression predictions of non-compliant behaviors would still be accurate in the direction. However, the effect sizes of predictors on non-compliance may be smaller than the actual influence on behavior. That is, although the most influential significant predictors would still be identified by the current analysis, other predictors may emerge as significant if a sample of known non-compliers only were engaged. Approaching people in crowded places and/or not wearing masks may allow a better assessment of predictors of those behaviors. Doing so, however, would put researchers at risk. It may be difficult to find individuals who do not engage in handwashing upon return to the home, as this is a non-public behavior performed in a private place. Of the behaviors, only the taking of antibiotics is likely to be approachable in a non-complier population, but would require permission from health care providers and pharmacists to identify individuals who sought out antibiotics to treat of prevent COVID-19. Overall, we believe that social desirability bias, however, is unlikely to impact the implications for practice from this study.

The study may also not fully represent Venezuelans. Our study over-represented women, older persons, and more educated persons in comparison to the population of Venezuela. Although there were sufficient representations of each demographic groupings to perform binomial logistic regressions, given potential overlaps among variables (such as younger people being more likely to be singletons and/or students) larger representations within each category would allow greater distinctions to be made between class members (e.g., sufficient married versus single young people who are students and compared to married versus single young people who are manual laborers to parse in greater detail to contributions of each variable). The participants in this study were also more likely to be socioeconomically advantaged, as indicated by the higher levels of education and smaller numbers of manual laborers. This may be due to the online methodology of recruiting. When face-to-face participation is safe for researchers and for participants, future studies should consider incept methodologies to complement internet methodologies.

## Conclusion

In summary, Venezuelans, as represented by this research, have fairly good levels of knowledge related to COVID-19, and better levels that in other samples from Colombia and Ecuador. Venezuelans are likely to practice recommended behaviors to prevent the further spread of COVID-19 and, as Venezuelans become more knowledgeable of COVID-19 they are more likely to adopt these practices. Venezuelans are optimistic about the world overcoming COVID-19, but pessimistic of Venezuela’s eventual success, suggesting an important focal point for messaging to promote optimistic attitudes that enable attitudes supportive of the control of COVID-19.
